# Nutritional assessment and serum zinc and copper concentration among children with acute lymphocytic leukemia: a longitudinal study

**DOI:** 10.1590/S1516-31802006000600003

**Published:** 2006-11-01

**Authors:** Ursula Rohr Sgarbieri, Mauro Fisberg, Luís Gonzaga Tone, Maria do Rosário Dias Latorre

**Keywords:** Leukemia, Child, Nutritional status, Zinc, Copper, Leucemia, Criança, Estado nutricional, Zinco, Cobre

## Abstract

**CONTEXT AND OBJECTIVE:**

When undergoing chemotherapy and/or radiotherapy, children with acute lymphocytic leukemia may present important nutritional disorders because of the gastrointestinal toxicity of most chemotherapy agents or the effects of radiation on the organism. These patients may also present changes in their serum concentrations of trace elements such as zinc and copper. The present study aimed to follow anthropometric parameters and serum levels of zinc and copper in a group of children under treatment for acute lymphocytic leukemia.

**DESIGN AND SETTING:**

Longitudinal study, at the Pediatric Section of Hospital das Clínicas, Ribeirão Preto, Brazil.

**METHODS:**

Forty-five children with acute lymphocytic leukemia were studied. Anthropometric parameters such as weight and height and the daily intakes and serum levels of copper and zinc were recorded at diagnosis and during the treatment.

**RESULTS:**

During the initial phase of the treatment, there was an increase in energy intake accompanied by weight gain. However, during the later phases of treatment there was a reduction in energy intake with accompanying weight loss. Decreased growth rate during treatment was more pronounced in children with high-risk acute lymphocytic leukemia, probably due to radiation therapy. Serum zinc levels remained basically unaltered during the treatment, whereas copper levels decreased dramatically with the beginning of treatment.

**CONCLUSIONS:**

The treatment given to children with acute lymphocytic leukemia has an important effect on their linear growth rate and nutritional status, and also on their serum copper levels.

## RESUMO

**CONTEXTO E OBJETIVO:**

Crianças portadoras de leucemia linfóide aguda quando em tratamento quimioterápico e/ou radioterápico podem apresentar comprometimento do estado nutricional devido aos efeitos tóxicos a nível gastrointestinal dos agentes quimioterápicos ou aos efeitos da radioterapia sobre o organismo. Esses pacientes também podem apresentar alterações nas concentrações séricas de zinco e cobre. O objetivo foi acompanhar os parâmetros antropométricos e os níveis séricos de zinco e cobre de crianças em tratamento para leucemia linfóide aguda.

**TIPO DE ESTUDO E LOCAL:**

Estudo longitudinal, realizado no Hospital das Clínicas da Faculdade de Medicina de Ribeirão Preto, Ribeirão Preto, São Paulo, Brasil, que envolveu 45 crianças do Serviço de Oncologia pediátrica, portadoras de leucemia linfóide aguda.

**MÉTODOS:**

Medidas antropométricas como peso e altura, medidas de consumo alimentar, assim como níveis séricos de zinco e cobre foram avaliados ao diagnóstico e durante o tratamento.

**RESULTADOS:**

No início do tratamento, houve aumento na ingestão calórica, acompanhada de ganho de peso, no entanto, nas fases seguintes ao tratamento houve redução na ingestão alimentar, resultando em perda de peso. Diminuição na velocidade de crescimento foi notada nas crianças com leucemia linfóide aguda de alto risco que foram submetidas a radioterapia. Os níveis séricos de zinco não sofreram alterações durante o tratamento, já os níveis séricos de cobre tiveram importante diminuição em relação ao início do tratamento.

**CONCLUSÕES:**

Pudemos observar alterações no estado nutricional, assim como alterações nas concentrações séricas de micronutrientes durante o tratamento para leucemia linfóide aguda.

## INTRODUCTION

Cancer, like other chronic diseases, can have an adverse effect on the nutrient balance because of a combination of factors.^1^ Children with cancer are at risk of suffering from undernutrition that, if severe, affects tolerance and may influence the patient's overall survival.^2^ Cancer or cancer therapy may result in anorexia, vomiting or maldigestion/malabsorption, with the net result of reductions in absorbed nutrient intake.^1^ However, controversy exists regarding the influence of chemotherapy or radiotherapy on nutritional status.^3^

Over the last decade, improved chemotherapy strategies for childhood leukemia have resulted in a dramatic improvement in survival rates, such that most children are now definitively cured.^4,5^The evolution of supportive care strategies has also contributed towards the advances obtained by chemotherapy intensification.^6^

Trace elements have been extensively studied over recent years, to assess whether they have any modifying effects regarding the etiology of cancer. Different authors have tried to establish a relationship between trace elements, especially zinc and copper, and malignant diseases. Changes in blood zinc and copper have been found in lymphoproliferative disorders, as well as in breast, lung and gastrointestinal tumors.^7-10^

The general trend towards slightly decreased zinc concentrations in malignant diseases supports the experimental results obtained by Brown et al.,^11^ thus suggesting that zinc deficiency is associated with the etiology of cancer.

Several studies^12,13^ have shown that serum copper levels in cases of malignant disease increase with increasing disease activity. Remission is usually associated with the return of copper levels to normal ranges. It has been suggested that serum copper would be a useful indicator for the extent of leukemia and malignant lymphoma, and might be a predictor for chemotherapy response.

## OBJECTIVE

The aim of this longitudinal study was to determine the nutritional status, growth pattern and serum zinc and copper concentrations at diagnosis and during treatment, among children with acute lymphocytic leukemia.

## METHODS AND MATERIALS

The study group comprised 45 children with newly diagnosed acute lymphocytic leukemia (ALL), between the ages of one and eleven years, who were admitted consecutively to the Pediatric Section of the Hospital das Clínicas, Ribeirão Preto, São Paulo, Brazil, between January 2000 and December 2002. The diagnosis was made by means of cytochemical stains and bone marrow smears. All the patients were enrolled in the study before receiving the first course of chemotherapy. The group consisted of 16 girls and 29 boys, and their median age was five years. The socioeconomic indicators for the children's families demonstrated that these children were mainly from low-income families.

All children were treated in accordance with the GBTLI-ALL protocol (Brazilian group for treatment of acute lymphocytic leukemia in infancy)14 and were classified into two groups according to this protocol, as follows:

- High-risk ALL (children aged less than 18 months or more than 10 years, or children with central nervous system involvement or with leukocyte counts > 50,000/mm^3^ at the time of diagnosis).
- Low-risk ALL (children aged between 18 months and 10 years, or children without central nervous system involvement or with leukocyte counts between 10,000 and 50,000/mm^3^ at the time of diagnosis).

The antineoplastic combined chemotherapy protocol comprised dexamethasone, daunorubicin, vincristine, L-asparaginase, citarabine and intrathecal methotrexate as the induction therapy. The reinduction treatment included vincristine, dexamethasone, asparaginase, citarabine and radiotherapy in children with high risk ALL. The maintenance treatment included prednisone, citarabine 6-mercaptopurine and L-asparaginase.

The protocol was approved by the ethics committee of Universidade de São Paulo, Ribeirão Preto, São Paulo, Brazil, and all the families were informed and accepted the protocol procedures.

### Nutritional assessment

Standardized anthropometric measurements of body weight (W) and height (H) were made by trained nurses. The measurements were expressed as Z scores for H/A and W/H (height/age and weight/height), which are the differences between the child's weight and height and the mean weight-for-height and height-for age according to the charts from the World Health Organization (WHO, 1986).^15^ Malnourished status was diagnosed when the indices were more than two standard deviations below the mean values expected according to the international growth reference.

Mean daily intakes were assessed by means of a 24-hour dietary recall,^16^ which was produced in conjunction with the child's mother. The findings were translated into calorie, protein, zinc and copper estimates and compared with the Recommended Dietary Allowance (RDA) and Dietary Recommended Intakes (DRI) nutritional standards.^17,18^

Blood samples were obtained by venous puncture, after overnight fasting. Atomic absorption spectrophotometry for biochemical evaluation of serum blood samples was performed in the pediatric oncology laboratory using a spectrophotometer (Perkin-Elmer model 380). The blood was centrifuged and stored at −20°C until analysis. Plasticware was used throughout the procedures, with careful elimination of external contamination. Serum metals were expressed as µg/dl and the normal levels were defined as 50-120 µg/dl for zinc and 70-120 µg/dl for copper.

The anthropometrics, daily intakes and serum trace elements were measured four times during the study period, i.e. at diagnosis and than during each therapy period (induction, reinduction and maintenance therapy). The mean follow-up period for all the children was 18 months.

### Statistical methods

The results were expressed as means ± standard deviations. Friedman's test for repeated measurements was used to compare data from the patients over the study period. p values < 0.05 were considered statistically significant.

## RESULTS

### At diagnosis:

The characteristics of this population of 45 patients at diagnosis are presented in Table 1. The anthropometric indices used for assessing the children's nutritional status demonstrated that five children appeared to be in the malnourished range. Three patients had a weight-for-height score of less than −2 and two had height-for-age score of less than −2.

**Table 1:** Characteristics of the children at diagnosis of acute lymphocytic leukemia (ALL)

**Total**	**45**
Male	29
Female	16
Age (year)	5 ± 2.6
High-risk ALL	16
Low-risk ALL	29
Z-score (W/H) ≤ −2	3
Z-score (H/A) ≤ −2	2

*W = weight; H = height; A = age.*

Reduced calorie intake was seen in 76% of the children. Changes in daily routine, hospitalization, aggressive medical procedures, some nonspecific symptoms (like anemia, anorexia and fatigue) and the psychological effects from the diagnosis were mentioned by parents as the main causes for changes in the children's dietary habits and low calorie intake.

Since the majority of the children started treatment with normal nutritional status, we believe that their low calorie intake had not been long enough to produce weight loss.

With regard to protein intake, we observed that only 9% of our children had intakes below the recommendations (RDA) Nevertheless, not only the quantity but also the quality of the protein needs to be taken into account. Reports from the Food and Agriculture Organization (FAO/WHO)^19^ have suggested that quality is influenced more by family income than is quantity. In our study, we observed that high protein intake was mainly due to frequent intake of rice with beans and milk.

For zinc and copper, 24% and 2% of the children had intakes below the dietary reference intake (DRI) standards, respectively.

The mean serum zinc concentration was 100.8 ± 25 µg/dl, and therefore within the normal range. The mean serum copper concentration was 206 ± 75 µg/dl, i.e. significantly above normal levels (90 µg/dl) (p < 0.05).

### During treatment

**Figure 1:**
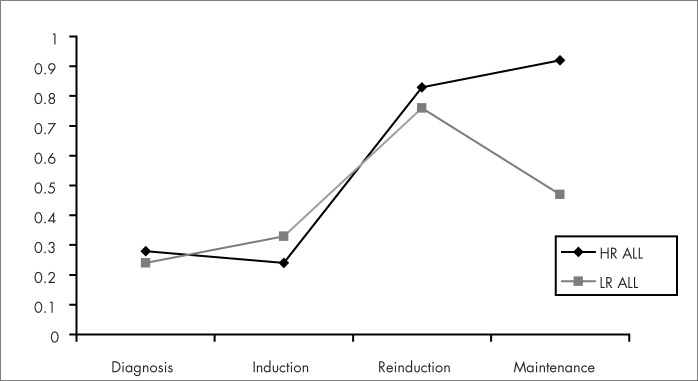
Mean Z-scores for weight-for-height at the stages of diagnosis, induction, reinduction and maintenance therapy in the groups of children with high-risk acute lymphocytic leukemia (HR ALL) and low-risk acute lymphocytic leukemia (LR ALL).

**Figure 2:**
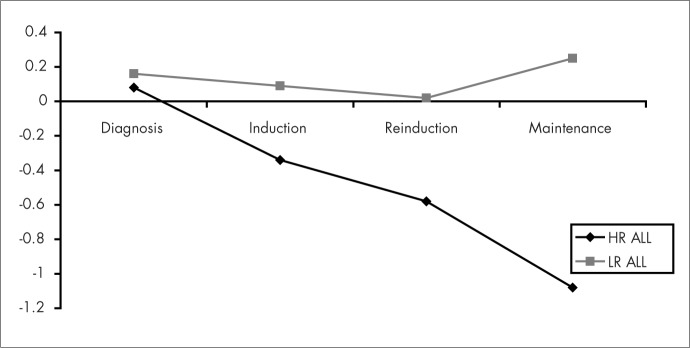
Mean Z-scores for height-for-age at the stages of diagnosis, induction, reinduction and maintenance therapy in the groups of children with high-risk acute lymphocytic leukemia (HR ALL) and low-risk acute lymphocytic leukemia (LR ALL).

During induction and reinduction chemotherapy, the children gained weight and this was probably due to the use of high-dose steroids given over a period of weeks. During maintenance chemotherapy, the children presented weight loss, and especially among the group of children with low-risk ALL, as shown in Figure 1. A decrease in mean growth rate during treatment was evident, especially among the children with high-risk ALL who were treated with prophylactic cranial irradiation, as demonstrated in Figure 2. However, no statistically significant difference between the two groups was found.

The dietary intakes during treatment showed that the children increased their intakes of calories, protein, zinc and copper during induction and reinduction therapy and decreased their intakes during maintenance chemotherapy.

**Figure 3:**
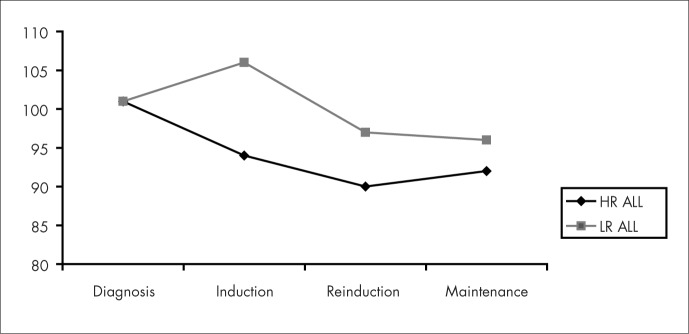
Serum zinc levels of the children with high-risk acute lymphocytic leukemia (HR ALL) and low-risk acute lymphocytic leukemia (LR ALL) at the stages of diagnosis, induction, reinduction and maintenance therapy.

**Figure 4:**
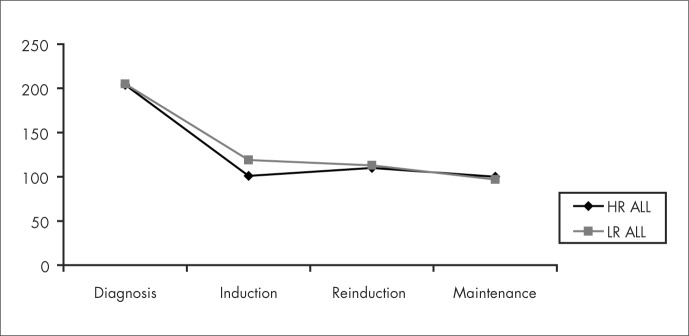
Copper levels in the children with high-risk acute lymphocytic leukemia (HR ALL) and low-risk acute lymphocytic leukemia (LR ALL) at the stages of diagnosis, induction, reinduction and maintenance therapy.

Serum zinc levels did not change significantly during treatment but serum copper levels decreased with the induction chemotherapy and remained stable during the rest of the treatment (Figures 3 and 4). No significant difference was found between the high-risk leukemia group and the low-risk leukemia group.

## DISCUSSION

Malnutrition has long been recognized as an important component of adverse outcomes among patients with cancer, including increased morbidity and mortality and decreased quality of life. The incorporation of nutritional screening and comprehensive assessments is increasingly recognized as imperative in the development of standards for quality care in oncology.^20^

A child with newly diagnosed cancer seems to have the same average nutritional status as is seen in the population from which the child comes, if the diagnosis is made in a reasonably timely manner.^21^ The incidence of malnutrition at diagnosis in our study was not as high as found by Viana et al.,^22^ who found that 21.2% of children with newly diagnosed ALL had a weight-for-age score of less than −2 and 17.4% had a height-for-age score of less than −2. We believe that the small number of malnourished children at diagnosis in our study is related to the improvement in socioeconomic factors seen in our country. The undernutrition indicators in Brazil have declined markedly and continuously since the mid-1980s and mid-1990s, among children and adults in all regions and income strata, and especially in the southeastern region (state of São Paulo), as described by Monteiro and others.^23,24^ Malnutrition is, however, a well recognized problem during treatment and is therefore generally considered to be a consequence of cancer therapy.^25^

Assessment of nutritional status is difficult because there is currently no "gold standard" for assessment. In addition, nutritional status is multidimensional and can be assessed by methods that are anthropometric, biochemical, dietary, clinical and functional.^26^ Weight, height and body mass index are used for most clinical purposes in pediatrics, and are particularly suitable for patients with ALL because height and weight are measured routinely and carefully.^27^

In our study we noticed that treatment produced changes in daily intake, in weight and height, and also in serum copper levels.

### Weight

Several factors may affect weight, including drugs, diet and lack of physical exercise. The induction and reinduction chemotherapy included the use of high-dose steroids given over a period of weeks, and our children gained weight rapidly during this time. Glucocorticoid treatment for childhood acute lymphocytic leukemia increases energy intake markedly, and this effect contributes towards the excessive weight gain and obesity that are characteristic of patients undergoing treatment for lymphocytic leukemia.^28^ Water retention in some of our children could also explain their weight gains. However, we observed that the children's calorie intake during induction and reinduction chemotherapy was 30% greater than at diagnosis. At that time, the children had an enormous desire to eat rice with beans (a typical Brazilian dish), meat, bread and pasta. Even during the night, the children were asking for meat, rice and beans. During the maintenance therapy period, we observed a decrease in energy and nutrient intake and some weight loss, probably due to the intermittent use of 6-mercaptopurine, since this drug produces an adverse effect on children's food intake.^29^

### Height

It is well known that growth retardation may occur during the treatment of children with acute lymphocytic leukemia.^30,31^ In our study, we observed growth retardation during treatment mainly among the children who received cranial irradiation. One cause of the growth retardation in patients with ALL is thought to be cranial irradiation, which affects growth hormone secretion, among other results.^32,33^ Cranial irradiation, however, cannot be the only cause of growth retardation. Cytostatic treatment with drugs like 6-mercaptopurine, methotrexate and vincristine could decrease production of insulin-like growth factor (IGF-I), and high doses of steroids also affect growth rate.^34^

The majority of children treated for ALL have significant changes in nutritional status, as manifested by reduced growth and alteration in body composition, with decreased lean mass and increased fat mass.

### Trace elements

The mean serum copper levels in our children at diagnosis were significantly higher than found in healthy children.^35^ Since many conditions can alter serum zinc and copper concentrations, several variables such as inflammatory conditions, infectious diseases and zinc and copper intakes were studied to determine their relative contributions. No positive correlations were found between these variables and blood concentrations.

The biological role of trace elements, especially of serum copper and zinc, in different physiological and pathological conditions has been extensively investigated over recent years. Changes in copper and zinc concentrations have been found in lymphoproliferative disorders and also in ovarian, breast, lung and gastrointestinal tumors.^8,9^ However, there is contradictory data regarding the degree of usefulness of copper and zinc for cancer diagnosis and prognosis.^36,37^

In our study, we observed altered concentrations of these two elements at diagnosis. With the beginning of treatment, we observed a notable decrease in copper concentrations, although during the rest of the study the levels remained unaltered. The zinc levels did not change significantly over the study period: they remained within the normal range but below the levels found in healthy children.

Measurements of circulating concentrations of ceruloplasmin and albumin/alpha-2 macroglobulin, which bind zinc and copper respectively, would have aided interpretation of the basal values and the changes over time.

We believe that blood determination of these two elements might be useful for estimating the presence of malignancies. However, as prognostic factors they did not seem very sensitive. A far more comprehensive study of the basic mechanisms for alteration of serum copper and zinc levels and their significance in relation to all malignancies is needed.

## CONCLUSIONS

Children treated for ALL present changes in nutritional status, as manifested by reduced growth, weight gains and weight losses. Serum copper and zinc levels may also be altered.

Since leukemia is the most common childhood cancer and is now a curable disease, it is important to identify alterations in nutritional status. Therefore, early and close attention to nutritional status is essential for an optimal outcome.

The changes in serum copper and zinc levels found in this study have been found in other lymphoproliferative disorders. However, further studies are necessary to better interpret these findings.
